# Supplementary material for: Birinapant Reshapes the Tumor Immunopeptidome and Enhances Antigen Presentation
Source: Int J Mol Sci. 2024 Mar 25;25(7):3660. doi: 10.3390/ijms25073660 (PMC11011986; doi:10.3390/ijms25073660)
Supplement: Supplementary file 1 [file ijms-25-03660-s001.zip › ijms-2883401-supplementary.pdf]

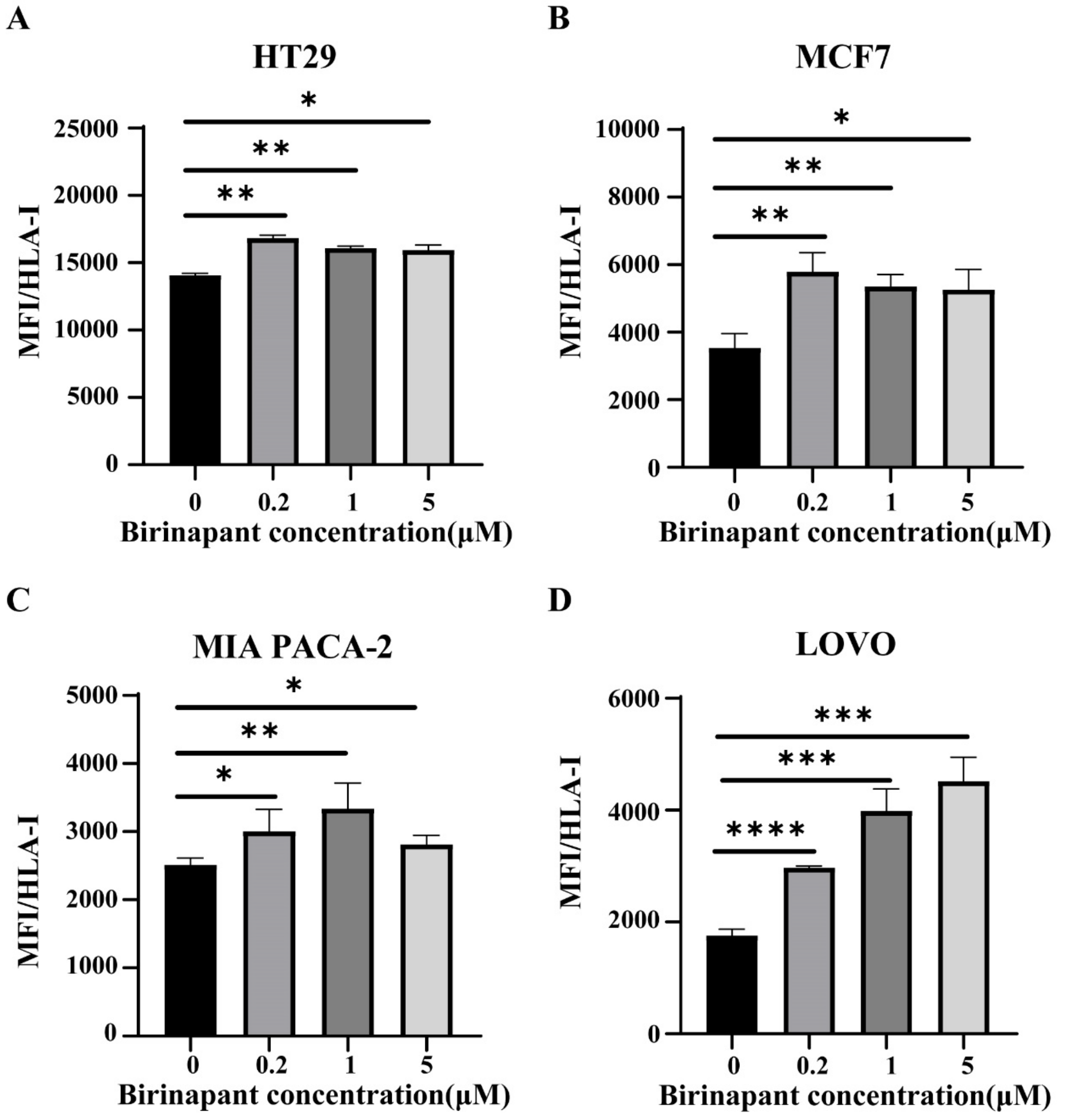

Figure S1 Concentration-effect of Birinapant on the expression level of HLA-I in different cell lines

Different concentrations of Birinapant (0.2 μM, 1 μM, 5 μM) were used to treat HT29 cells (A), MCF7 cells (B), MIA-PACA-2 cells (C), and LOVO cells (D). Each experiment was repeated at least three times, and the median fluorescence intensity (MFI) of HLA-I was calculated. Noteworthy, it was inappropriate to compare MFI among cell lines for data obtained at different times, since MFI may be influenced by instrument conditions. In HT29 and MCF7 cells, the highest MFI was seen at a Birinapant concentration of 0.2, which directed subsequent experiments. *: $p<0.05$; **: $p<0.005$; ***: $p<0.0005$; ****: $p<0.0001$.

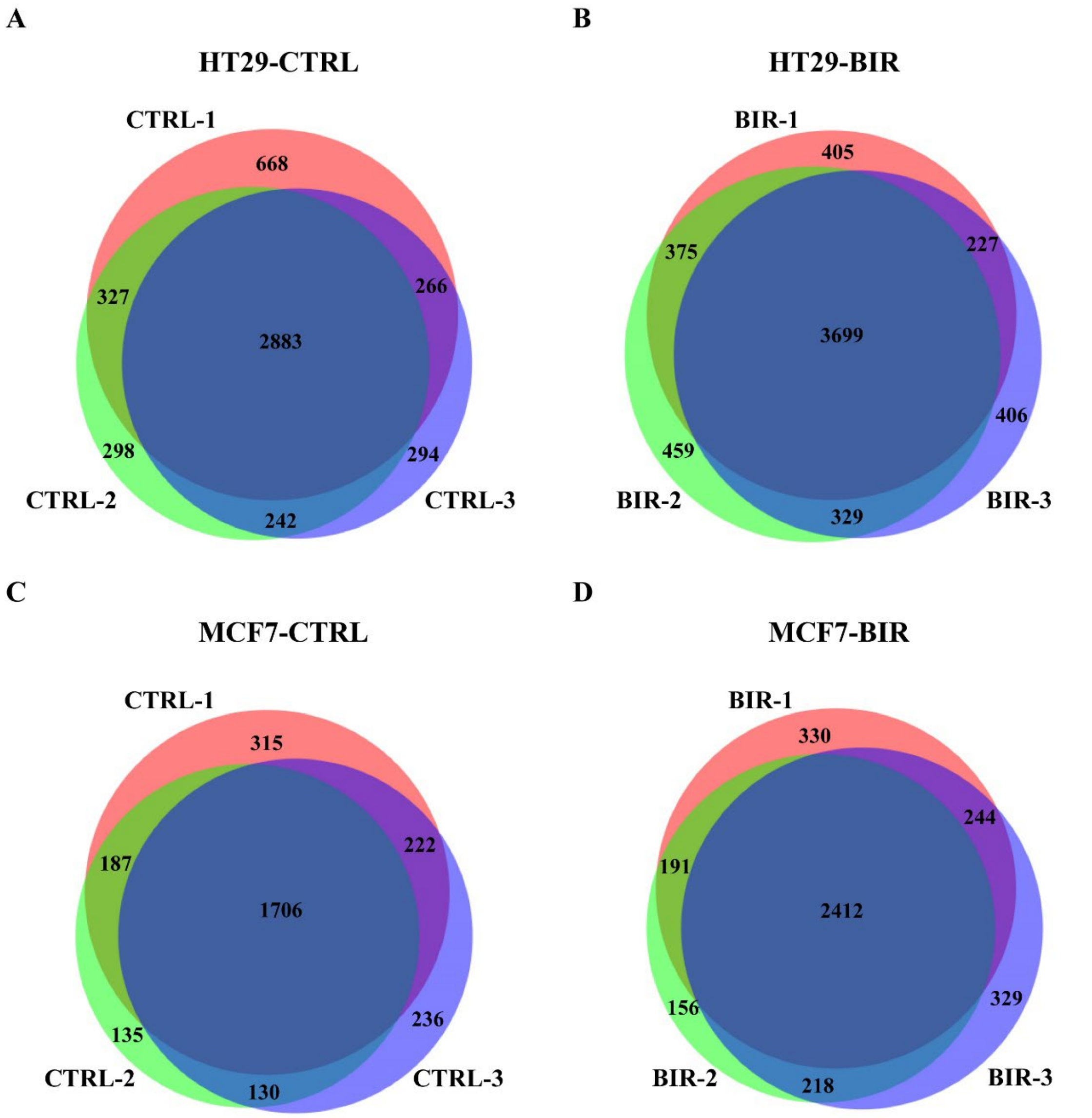

Figure S2 Immunopeptide distribution of triplicate samples of HT29 and MCF7

Overlap between triplicate samples of HT29-CTRL (A), HT29-BIR (B), MCF7-CTRL (C), and MCF7-BIR (D). (A) Specifically, 4,144, 3,750, and 3,685 peptides were identified in HT29-CTRL; 2,883 were shared and 3,718 were identified at least twice. (B) A total of 4,706, 4,862, and 4,661 peptides were identified in HT29-BIR; 3,699 were shared and 4,630 were identified at least twice. (C) A total of 2,430, 2,158, and 2,294 peptides were identified in MCF7-CTRL; 1,706 were shared and 2,430 were identified at least twice. (D) A total of 3,177, 2,977, and 3,203 peptides were identified in MCF7-BIR; 2,412 were shared and 3,065 were identified at least twice.

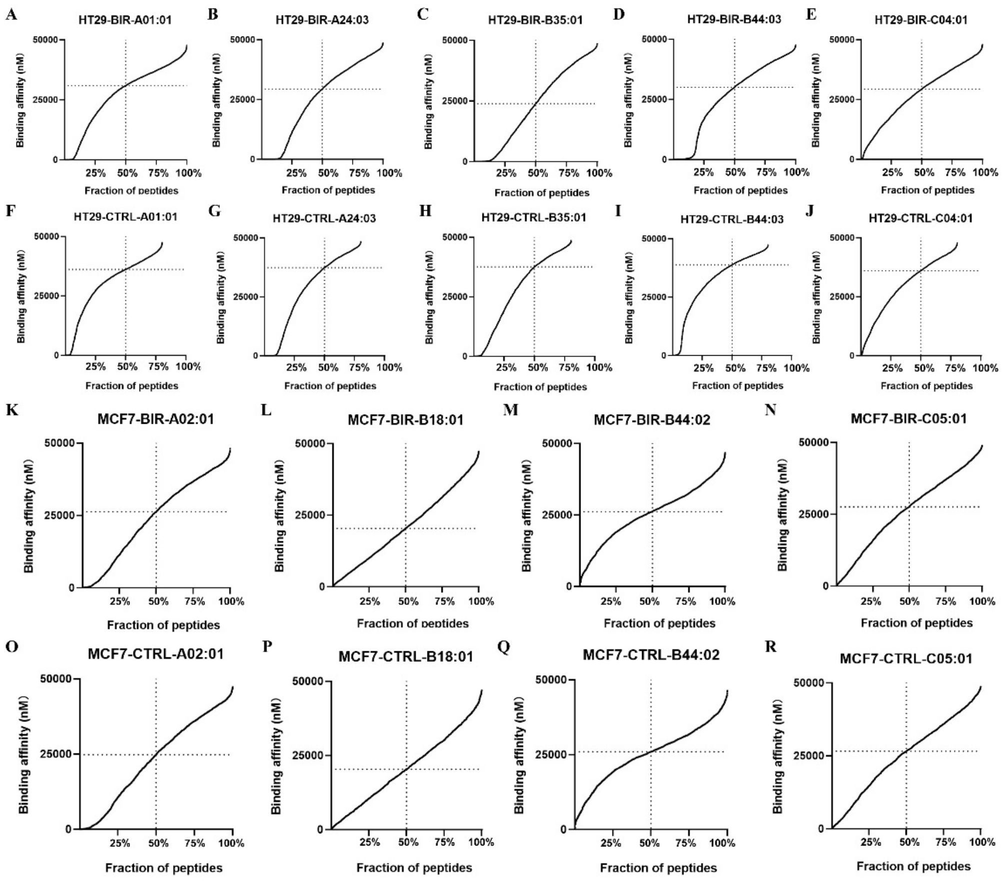

Figure S3 Predicted binding affinity distribution of HT29 and MCF7.

Predicted binding affinity (BA) was obtained from netMHCpan4.1, for each HLA allele, detected the corresponding BA of all peptides and list them from high to low. Before and after Birinapant treatment, the basic feature of BA distribution was similar, while the median number of BA had difference.

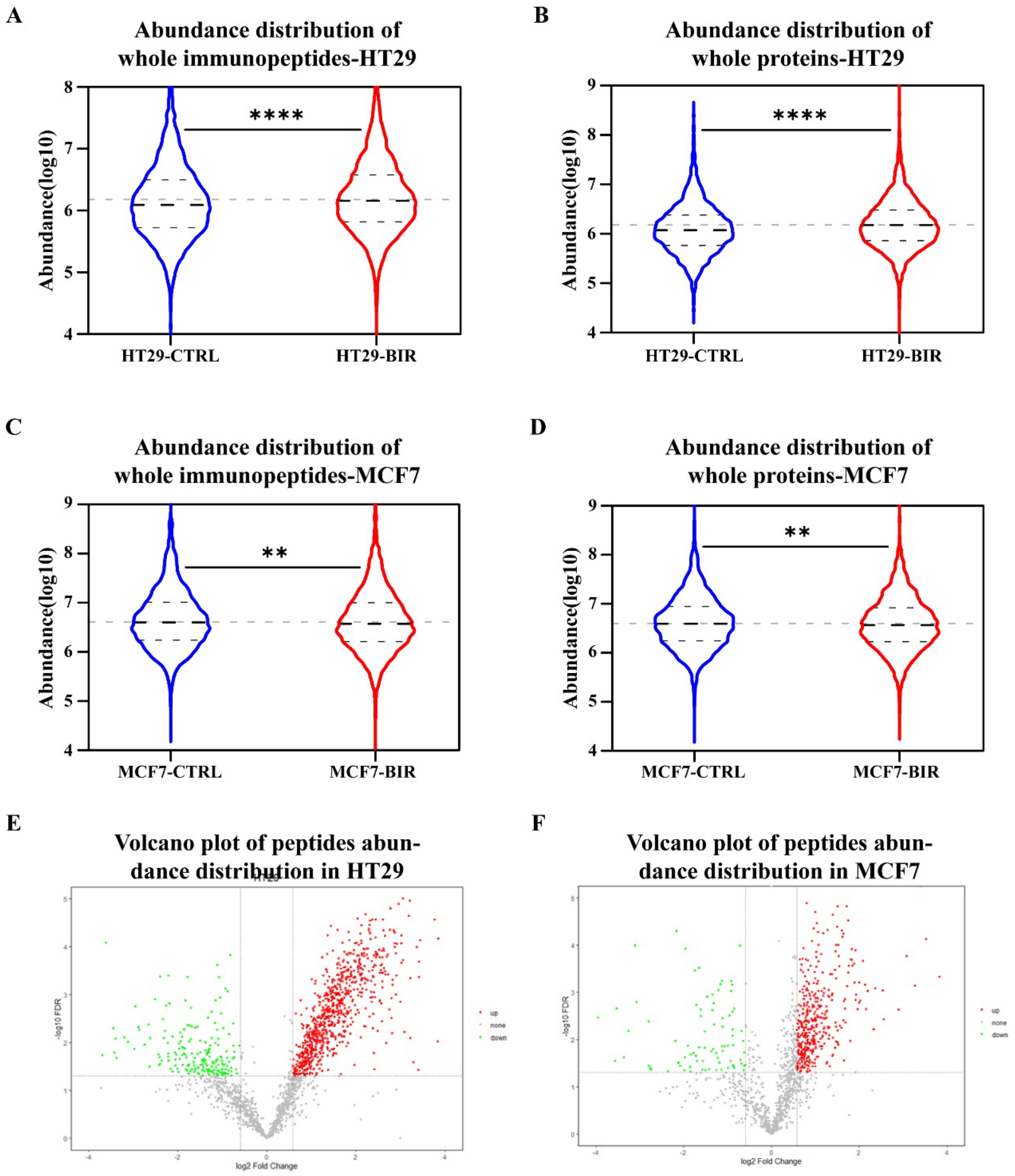

Figure S4 Abundance distribution of whole proteins in HT29 and MCF7

Label-free quantitation (LFQ) was used to obtain the abundance of immunopeptides and their source proteins. (A) In HT29 cells, the average abundance of immunopeptides was 6.14 (CTRL) and 6.22 (BIR). (B) In HT29 cells, the average abundance of source proteins was 6.09 (CTRL) and 6.19 (BIR). (C) In MCF7 cells, the average abundance of immunopeptides was 6.66 (CTRL) and 6.63 (BIR). (D) In MCF7 cells, the average abundance of source proteins was 6.62 (CTRL) and 6.59 (BIR). Although the abundance of peptides or proteins was lower in BIR in MCF7 cells, we suppose that specific peptides in BIR had a lower abundance, thus showing a lower overall abundance. (E) Use volcano plot to show abundance variation of peptides in HT29, and 825 peptides showed 1.5-fold increase, 199 peptides showed 1.5-fold decrease. (F) Use volcano plot to show abundance

variation of peptides in MCF7, and 425 peptides showed 1.5-fold increase, 86 peptides showed 1.5-fold decrease. **: $p<0.005$; ****: $p<0.0001$.

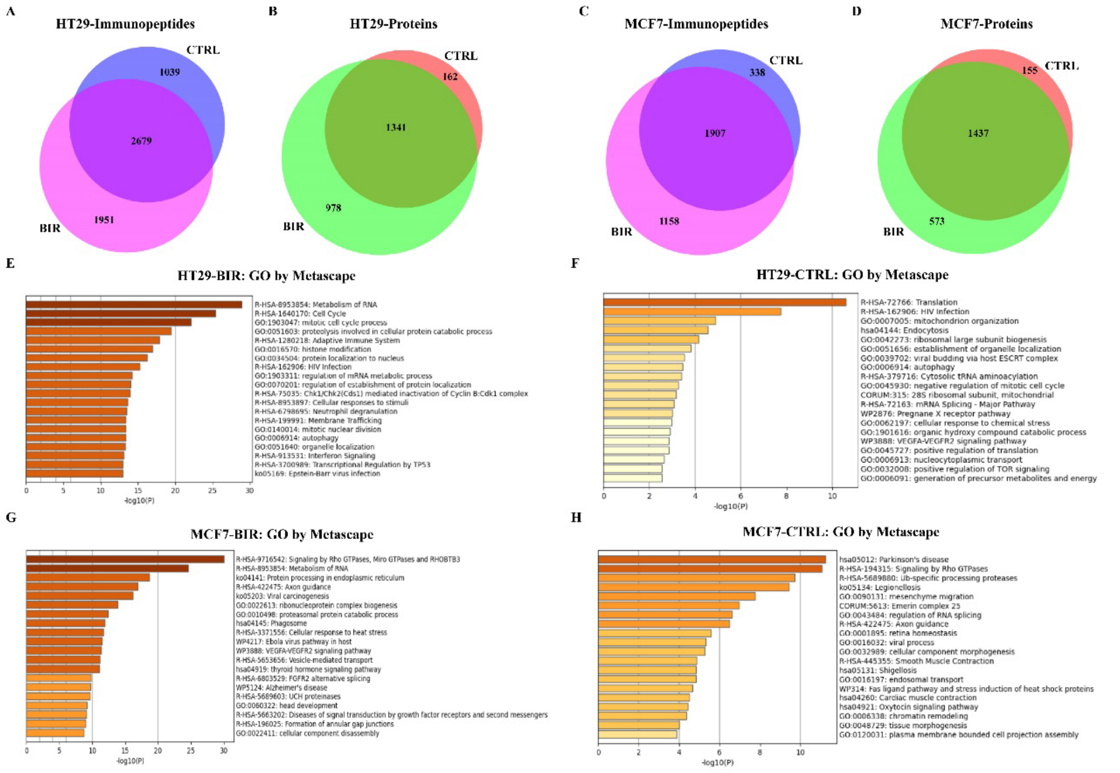

Figure S5. Functional clustering for source proteins of the immunopeptidome. (A) In HT29 cells, 72.05% of immunopeptides (2,679/3,718) in CTRL reappeared in BIR, while an extra 1,951 immunopeptides were specific to BIR. (B) In HT29 cells, 3,718 immunopeptides in CTRL were derived from 1,503 proteins, 4,630 immunopeptides in BIR were derived from 2,319 proteins, and 89.22% (1,341/1,503) were shared. (C) In MCF7 cells, 84.94% of immunopeptides (1,907/2,245) in CTRL reappeared in BIR, and these immunopeptides were derived from 1,592 proteins; 3,065 immunopeptides were derived from 2,010 proteins in BIR (D), and 90.26% (1,437/1,592) were shared. (E) Gene ontology (GO) terms for specific proteins in HT29-BIR according to Metascape (https://metascape.org). "Metabolism of RNA," "Cell cycle," and "Cellular protein catabolic processes" occupied the top positions. (F) By contrast, GO terms for specific proteins in HT29-CTRL showed different clusters with lower p-values. (G) GO terms for specific proteins in MCF7-BIR. "Metabolism of RNA" and "Protein processing" occupied the top positions, which is consistent with HT29-BIR. (H) GO terms for specific proteins in MCF7-CTRL. "Signaling by Rho GTPases" appeared in both MCF7-CTRL and MCF7-BIR, indicating that it is not a specific pathway affected by Birinapant.

Birinapant enhances antigen presentation

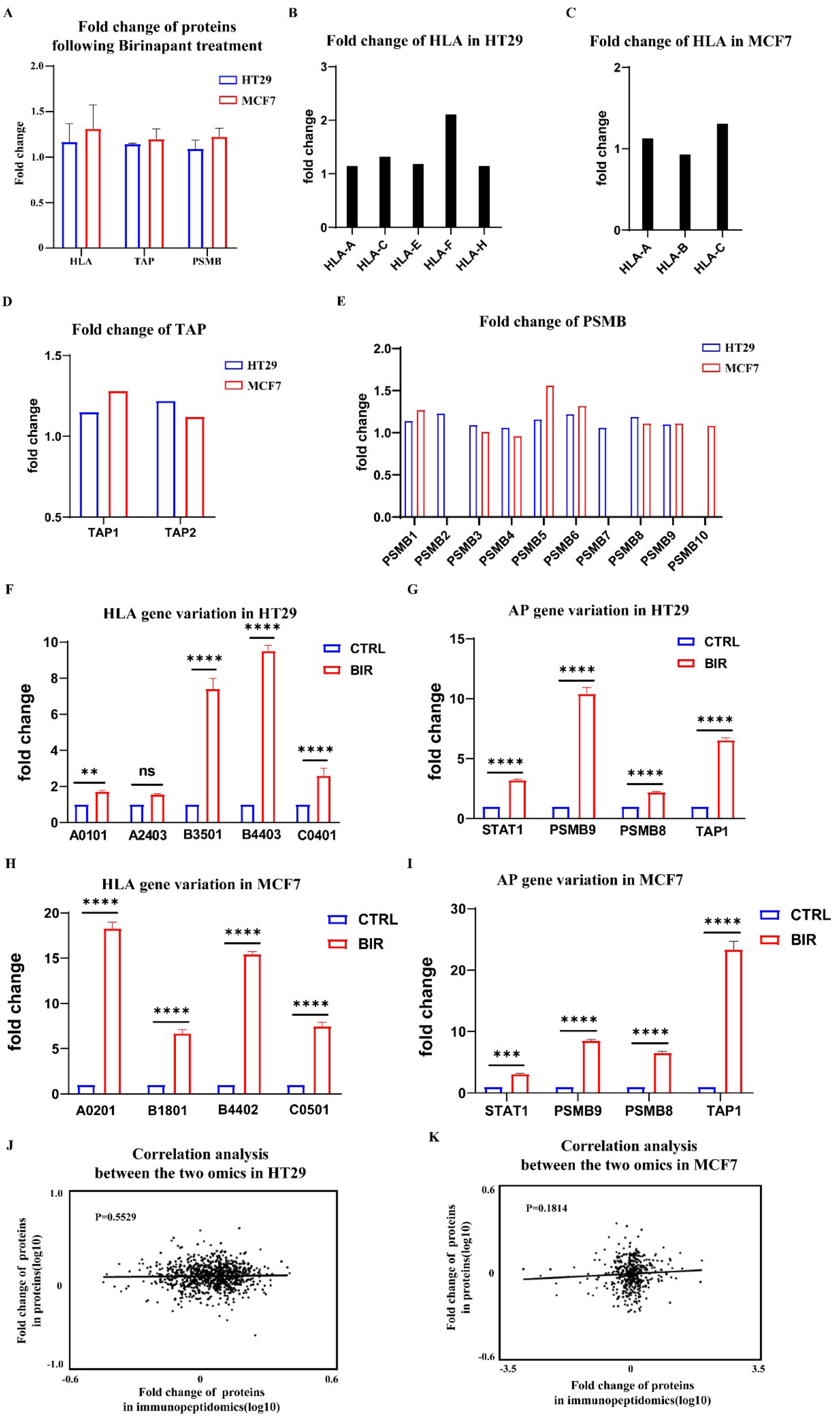

Figure S6 Comparison between the proteome and immunopeptidome

Sixplex tandem mass tag (TMT)-based quantitative proteomics was used to analyze abundance variation in proteins before and after Birinapant treatment. Six samples were assessed, three CTRL and three BIR. (A) Fold change in antigen presentation pathway-related proteins in the proteome. HLA-I was upregulated by an average of 1.30 times, TAP was upregulated by 1.15 times, and proteasome subunit beta (PSMB) was upregulated by 1.16 times. (B) Fold change of specific HLA genes in proteome of HT29. (C) Fold change of specific HLA genes in proteome of MCF7. (D) Fold change of specific TAP genes in proteome of two cells. (E) Fold change of specific PSMB genes in proteome of two cells. (F) Fold change of HLA gene in HT29 by QPCR. (G) Fold change of antigen presentation gene in HT29 by QPCR. (H) Fold change of HLA gene in MCF7 by QPCR. (I) Fold change of antigen presentation gene in MCF7 by QPCR. Screening proteins that could be quantitated in the proteome and immunopeptidome: 868 proteins in HT29 cells (J) showed little correlation between the two omics ($p = 0.5529$), and 434 proteins in MCF7 cells (K) confirmed this conclusion ($p = 0.1814$). ****: $p<0.0001$.

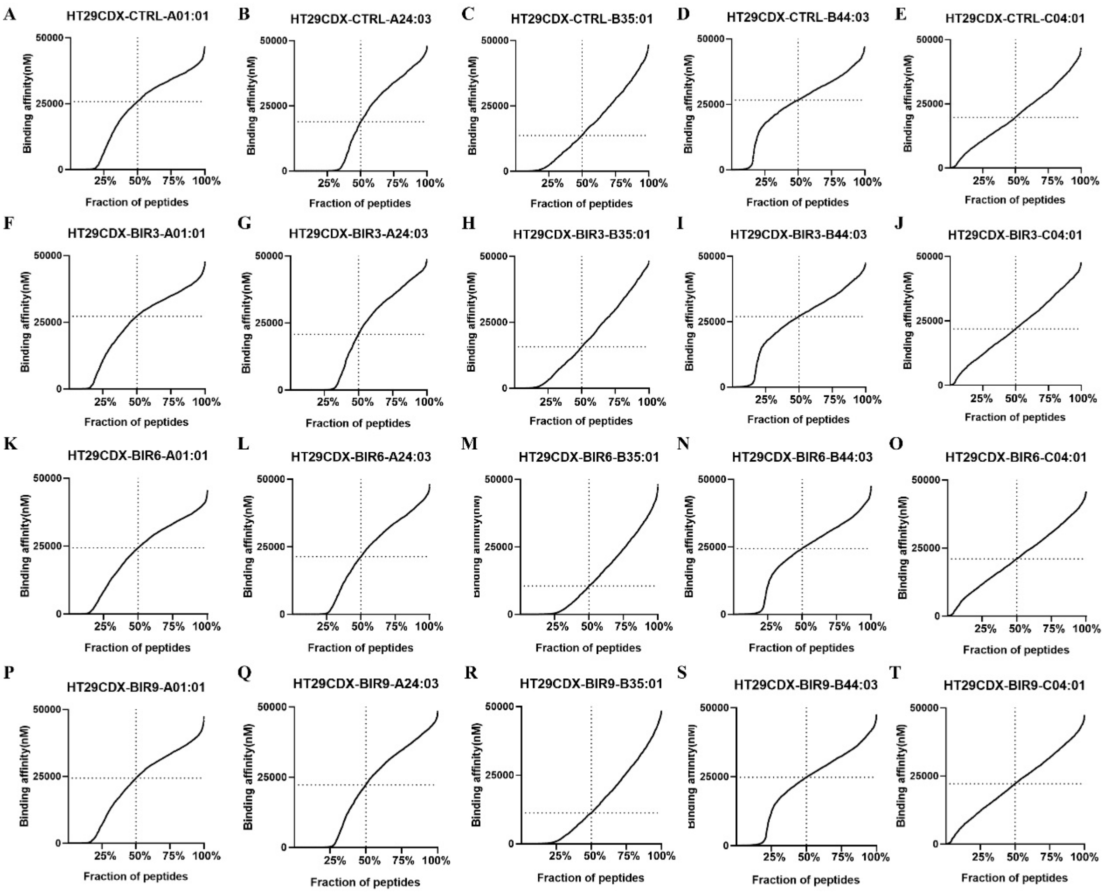

Figure S7 Predicted BA distribution of CDX.

Predicted BA was obtained from netMHCpan4.1, for each HLA allele, detected the corresponding BA of all peptides and list them from high to low. Before and after Birinapant treatment, the basic feature of BA distribution was similar, while the median number of BA had difference.

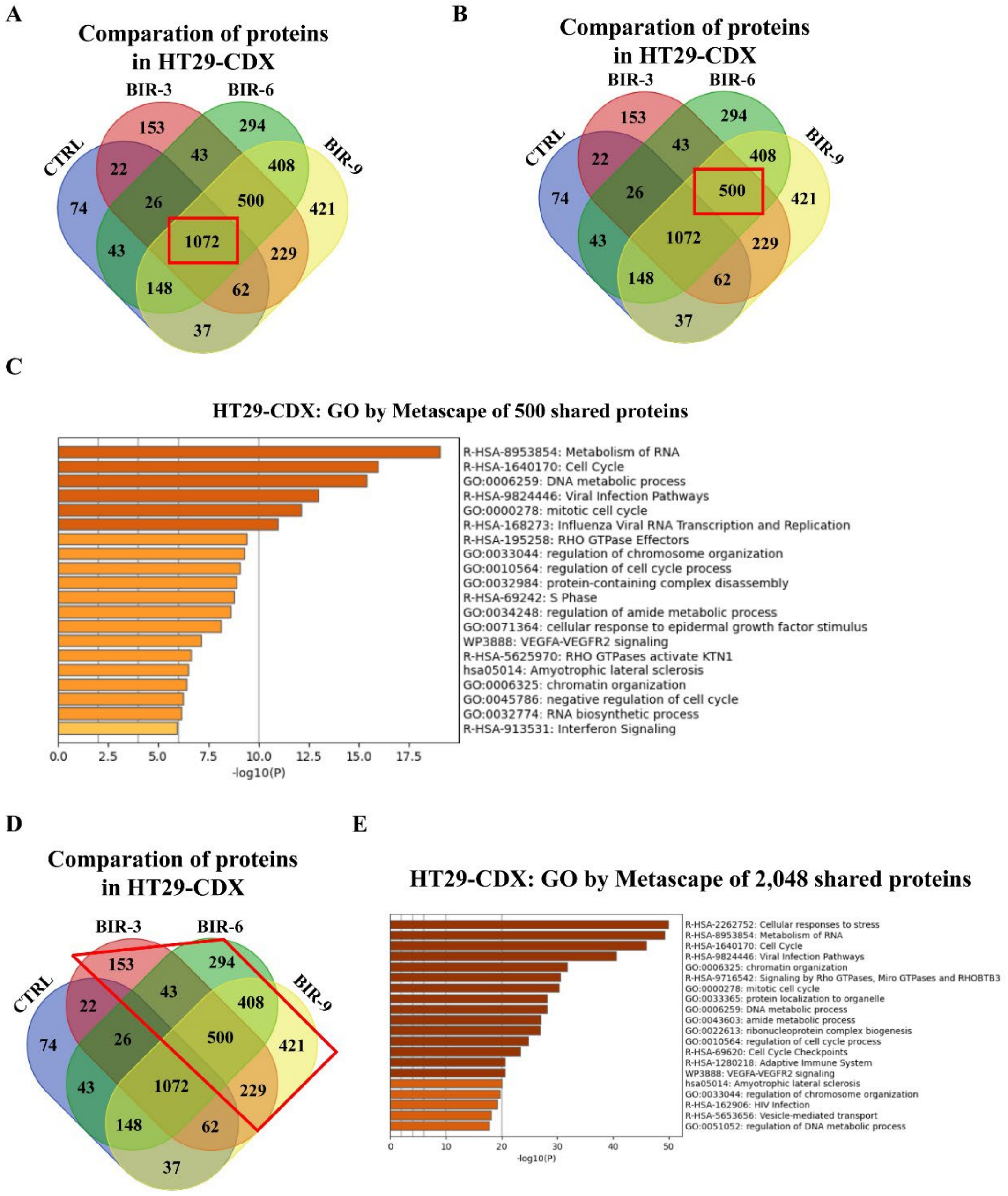

Figure S8 Comparison of immunopeptides and proteins in HT29-CDX

(A) Comparison of source proteins in HT29-CDX; 1,072 proteins were shared among the four groups. (B) Comparison of source proteins in HT29-CDX; 500 proteins were identified in BIR-3, BIR-6, and BIR-9 but not in CTRL. (C) GO terms for shared proteins (500 proteins, see Figure S8B) still clustered in "Metabolism of RNA" and "Cell Cycle," which is consistent with the clusters in BIR in HT29 cells. (D) Comparison of source proteins in HT29-CDX. Proteins circled in red were only identified in the three BIR groups and not in CTRL. A total of 2,048 proteins met this standard. (E) The 2,048 proteins in G were clustered in "Metabolism of RNA" and "Cell Cycle," which is consistent with the 500 proteins shared among the four groups (D). Meanwhile, there was an extra cluster related to "Cellular responses to stress" following prolonged Birinapant treatment time. This cluster was present again among the 421 specific proteins in BIR-9 (data not shown).

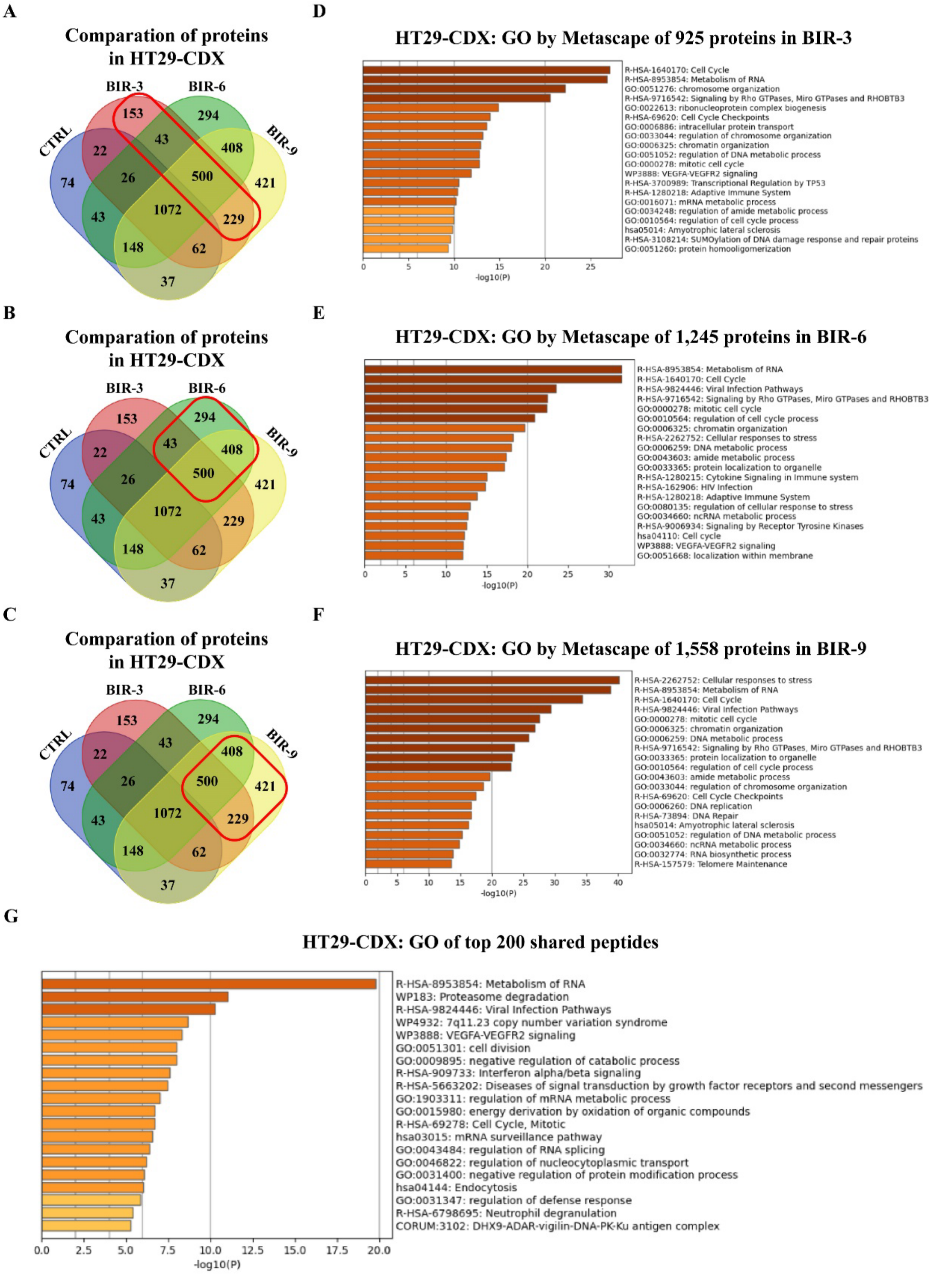

Figure S9 GO terms for specific proteins in different BIR groups

(A) Comparison of source proteins in HT29-CDX; 925 proteins were specific to BIR-3 in comparison with CTRL. Similarly, 1,245 proteins were specific to BIR-6 (B) and 1,558 proteins were specific to BIR-9 (C) in comparison with CTRL. (D) The 925 proteins specific to BIR-3 were clustered in "Metabolism of RNA" and "Cell Cycle," which is consistent with this conclusion. The 1,245 proteins specific to BIR-6 (E) and the 1,558 proteins specific to BIR-9 (F) were also present

in these two clusters. Although there were other clusters that appeared in the three groups at the same time, for example, "chromatin organization," we did not focus heavily on these since they were not always at the top positions. (G) The top 200 peptides were chosen according to the average ranking. Gene ontology (GO) terms for source proteins still clustered in "Metabolism of RNA," which is consistent with the GO terms for shared proteins, further indicating that Birinapant influences antigen presentation in specific pathways.

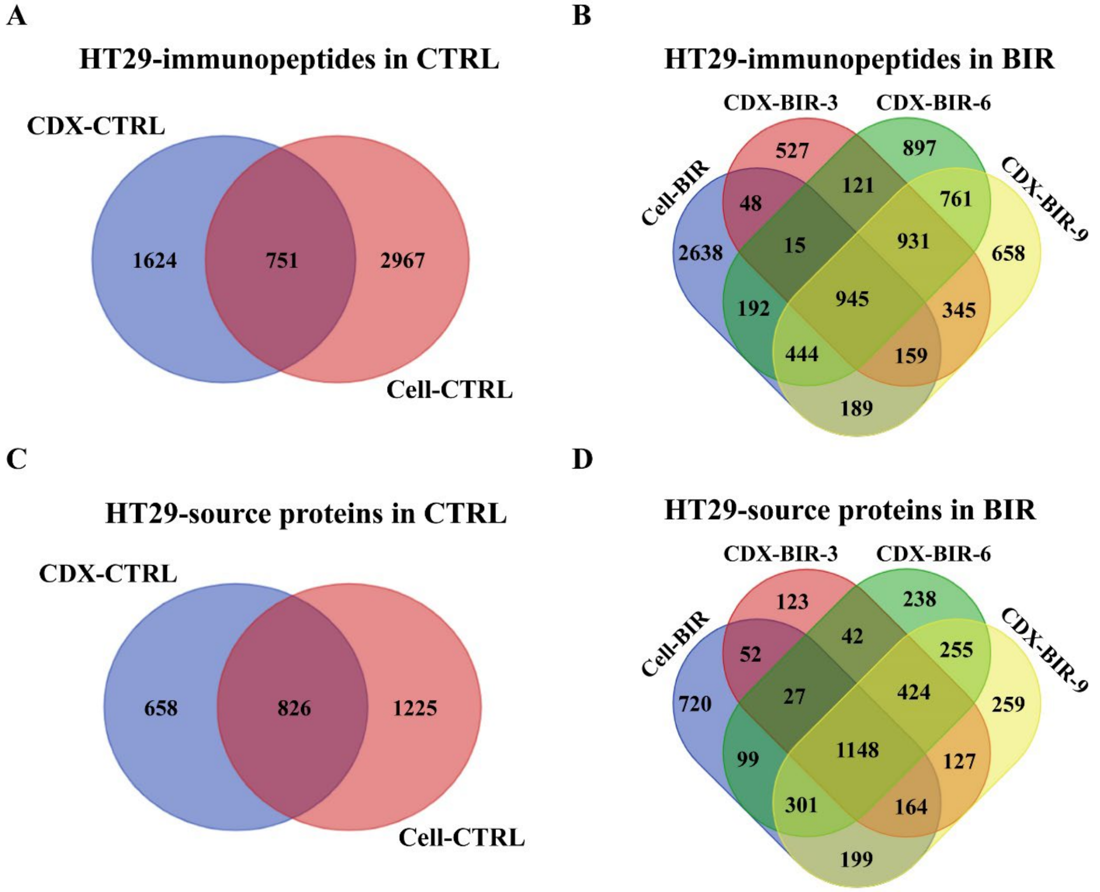

Figure S10 Comparison between the *in vitro* and *in vivo* immunopeptidome of HT29 cells

(A) For immunopeptides identified in CTRL (3,718 in Cell-CTRL and 2,375 in CDX-CTRL), only 751 were shared (20.20%, 751/3,718). (B) For immunopeptides identified in BIR (4,630 in Cell-BIR, 3,091 in CDX-BIR-3, 4,306 in CDX-BIR-6, and 4,432 in CDX-BIR-9), the percentage of shared peptides was 25.21% (1,167/4,630, Cell-B IR compared with CDX-BIR-3), 34.47% (1,597/4,630, Cell-BIR compared with CDX-BIR-6), and 37.52% (1,737/4,630, Cell-BIR compared with CDX-BIR-9). The overlap of immunopeptides between cells and CDX was not high. (C) For source proteins in CTRL, 40.27% (826/2,051) were shared. (D) In BIR, 2,710, 2,107, 2,534, and 2,877 proteins were identified in Cell-BIR, CDX-BIR-3, CDX-BIR-6, and CDX-BIR-9, respectively. The shared percentage was 51.33% (1,391/2,710, Cell-BIR compared with CDX-BIR-3), 58.12% (1,575/2,710, Cell-BIR compared with CDX-BIR-6), and 66.86% (1,812/2,710, Cell-BIR compared with CDX-BIR-9). Although the percentages of proteins were higher than those of peptides, they were still lower than the shared percentage between CTRL and BIR in cells or CDX.

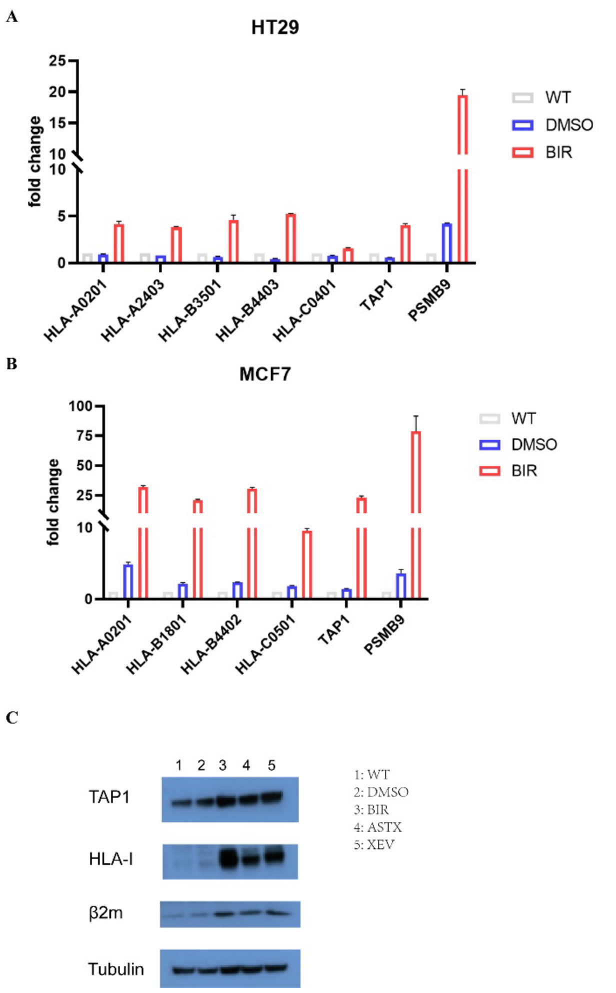

Figure S11 Effect of DMSO and SMAC mimetics on HLA-I expression.

Compared with BIR, DMSO showed negligible influence on HLA-I, TAP1 and PSMB9 (a subunit of immunoproteasome), both in HT29(A) and MCF7(B). (C) Western blot of MCF7 showed similarly results, ASTX and XEV are other two SMAC mimetics.

Table S1 HLA typing of PBMCs

Sample	HLA-A	HLA-B	HLA-C
HT29	01:01; 24:03	35:01; 44:03	04:01
PBMC	01:01; 02:01	07:02; 35:01	04:01; 07:02

(Tips: PBMC were used for validation the neoantigens identified in HT29, the matching HLA-I alleles in corresponding samples were marked in red.)
